# (Pro)renin receptor (ATP6AP2) depletion arrests As4.1 cells in the G0/G1 phase thereby increasing formation of primary cilia

**DOI:** 10.1111/jcmm.13069

**Published:** 2017-02-19

**Authors:** Heike Wanka, Philipp Lutze, Doreen Staar, Barbara Peters, Anica Morch, Lukas Vogel, Ravi Kumar Chilukoti, Georg Homuth, Jaroslaw Sczodrok, Inga Bäumgen, Jörg Peters

**Affiliations:** ^1^ Department of PhysiologyUniversity Medicine GreifswaldKarlsburgGermany; ^2^ Interfaculty Institute for Genetics and Functional GenomicsUniversity Medicine and Ernst Moritz Arndt‐University GreifswaldGreifswaldGermany

**Keywords:** (P)RR/ATP6AP2, cell cycle, ciliogenesis, proliferation, V‐ATPase

## Abstract

The (pro)renin receptor [(P)RR, ATP6AP2] is a multifunctional transmembrane protein that activates local renin–angiotensin systems, but also interacts with Wnt pathways and vacuolar H^+^‐ATPase (V‐ATPase) during organogenesis. The aim of this study was to characterize the role of ATP6AP2 in the cell cycle in more detail. *ATP6AP2* down‐regulation by siRNA in renal As4.1 cells resulted in a reduction in the rate of proliferation and a G0/G1 phase cell cycle arrest. We identified a number of novel target genes downstream of *ATP6AP2* knock‐down that were related to the primary cilium (*Bbs‐1, Bbs‐3, Bbs‐7, Rabl5, Ttc26, Mks‐11, Mks‐5, Mks‐2, Tctn2, Nme7*) and the cell cycle (*Pierce1, Clock, Ppif*). Accordingly, the number of cells expressing the primary cilium was markedly increased. We found no indication that these effects were dependent of V‐ATPase activity, as *ATP6AP2* knock‐down did not affect lysosomal pH and bafilomycin A neither influenced the ciliary expression pattern nor the percentage of ciliated cells. Furthermore, ATP6AP2 appears to be essential for mitosis. ATP6AP2 translocated from the endoplasmatic reticulum to mitotic spindle poles (pro‐, meta‐ and anaphase) and the central spindle bundle (telophase) and *ATP6AP2* knock‐down results in markedly deformed spindles. We conclude that ATP6AP2 is necessary for cell division, cell cycle progression and mitosis. ATP6AP2 also inhibits ciliogenesis, thus promoting proliferation and preventing differentiation.

## Introduction

The (P)RR is essential for life. Ablation of the (P)RR‐encoding gene, *ATP6AP2* (ATPase H^+^‐transporting lysosomal accessory protein 2), is lethal in zebrafish 1 and mice 2. Tissue‐specific ablation of ATP6AP2 results in end‐organ damage with heart failure 3 or renal failure 4. Mutations in ATP6AP2 are a cause of X chromosome‐linked mental retardation and epilepsy 5 and of X chromosome‐linked parkinsonism with spasticity in humans 6.

Although originally described as a transmembrane surface receptor that increases (pro)renin activity and hence local extracellular angiotensin production 7, there are intracellular functions of ATP6AP2 which are (pro)renin‐dependent but angiotensin‐independent. Such ATP6AP2 functions involve activation of both the extracellular signal‐regulated kinases 1 and 2 (ERK)/mitogen‐activated protein kinase pathway 7 and the transcription factor promyelocytic leukaemia zinc finger 8, 9. Newly discovered functions of ATP6AP2 are completely independent of the renin–angiotensin system, such as its effects on Wingless‐type (Wnt) pathways and V‐ATPase activity.

ATP6AP2 is intracellularly cleaved into an 8.9 and a 28‐kD fragment by furin or ADAM19 proteases. The M8.9 fragment of ATP6AP2 acts as an accessory subunit of V‐ATPase 10. The remaining 28‐kD fragment is secreted into the extracellular space 7, 11. Zebrafish with ATP6AP2 mutations share common embryonic phenotypes with mutants for different V‐ATPase subunits such as abnormal pigmentation, necrosis in the central nervous system, multi‐organ defects or lethality 1, 12.

Furthermore, ATP6AP2 was shown to function as an adaptor protein between V‐ATPase and the Wnt receptor complex in acidic endosomal compartments 12, 13. ATP6AP2 binds to the low‐density lipoprotein receptor‐related protein 6 (LRP6), Frizzled, and to distinct subunits of the V0 domain of the V‐ATPase, thereby modulating canonical Wnt/ß‐catenin signalling 12, 14. ATP6AP2 also contributes to the non‐canonical Wnt pathways [planar cell polarity (PCP), Ca^2+^] 13, 14. *ATP6AP2* silencing led to impaired targeting of the Wnt receptors Frizzled and Flamingo to the plasma membrane, implicating that ATP6AP2 may be a PCP core protein.

Previously, we have shown that ATP6AP2 is an essential component of the canonical Wnt pathway in adult neuronal stem cells, maintaining proliferation 12, 14. In contrast, when those cells differentiate, ATP6AP2 becomes a component of the non‐canonical Wnt/PCP pathway, which is essential for proper morphogenesis 12, 14. To date, it is unknown which steps of the cell cycle are affected by ATP6AP2. According to the function of the canonical Wnt pathway 15, we suggested that ATP6AP2, as part of this pathway, (*i*) should promote the progression from G1 to S phase, (*ii*) should stimulate proliferation by inducing the disassembly of the primary cilia and (*iii*) may be involved in spindle formation. As ATP6AP2 has been suggested to activate V‐ATPase activity 3, thereby taking part in the acidification of several cellular compartments, we tested the hypothesis whether acidification of lysosomal/endosomal compartments is involved in ATP6AP2 functions.

## Materials and methods

### Cell culture

As4.1 cells (ATCC, Manassas, VA, USA) were grown in DMEM medium (Lonza, Basel, Swiss) supplemented with 10% foetal bovine serum (PAN Biotech, Darmstadt, Germany), 100 U/ml penicillin and 100 μg/ml streptomycin (GIBCO, Life Technologies, Darmstadt, Germany) in a humidified incubator at 5% CO_2_ and 37°C.

### RNA interference and drug administration

10^5^ cells/2 ml medium were preincubated in six‐well plates for 2 days to reach 80% cell density before down‐regulation of ATP6AP2 or incubation with bafilomycin 1A (Enzo Life Science, Lörrach, Germany). For microscopy, 10^4^ cells/0.7 ml medium were seeded in four‐chamber cover slides and preincubated for 2 days.

For *ATP6AP2* knock‐down, transfection was performed for 6 hrs with a siGENOME SMART pool siRNA against *ATP6AP2* mRNA or scrambled control siRNA (Thermo Fisher Scientific Inc, Schwerte, Germany) in a final concentration of 40 nmol/l using Metafectene Pro (Biontex, Planegg/Martinsried, Germany) as transfection reagent. Time‐dependent down‐regulation was validated by qRT‐PCR and Western blot analyses.

Bafilomycin 1A was added to the cells for 1 day in a final concentration of 1 μmol/l. For these experiments, an additional control with 1% DMSO was used.

### Western blotting

Cells were extracted with lysis buffer containing 33.3 mM Tris, 3.33 mM EDTA, 100 mM NaCl, 6.67 mM K_2_HPO_4_, 6.67% glycerol, 0.033% SDS, 0.67% Triton X‐100, 1 mM NaVO_4_, 20 mM NaF, 0.1 mM PMSF, 20 mM 2‐phosphoglycerate and a protease inhibitor cocktail (Roche Diagnostics, Mannheim, Germany). Alternatively, to enrich specific cell fractions, cell membranes were cracked by digitonin buffer containing 150 mM NaCl, 50 mM HEPES, 25 μg/ml digitonin, 1 mM DTT, 0.5 mM PMSF and 5 mg/ml complete™ mini EDTA‐free (25×). Following incubation in digitonin buffer for 10 min. at 4°C, treated cells were centrifuged at 9300 ×*g* for 5 min. at 4°C. The supernatant, equivalent to the cytosolic fraction, was removed and stored at −20°C. The cell pellet was washed in phosphate‐buffered saline (PBS) and then incubated for 30 min. at 4°C in NP‐40 buffer containing 150 mM NaCl, 50 mM HEPES, 0.5 mM PMSF, 1% nonidet P40 (NP‐40), 1 mM DTT and 5 mg/ml complete™ mini EDTA‐free (25×). After centrifugation at 9300 ×*g* for 5 min. at 4°C, the supernatant containing membranes and different organelles was stored at −20°C. To obtain the nuclear fraction, the remaining cell pellet was washed in PBS and incubated for 1 hr at 4°C with lysis buffer containing 150 mM NaCl, 50 mM HEPES, 0.5% sodium desoxycholate, 0.1% SDS, 1 mM DTT, 0.5 mM PMSF, 1 U DNAse I and 5 mg/ml complete™ mini EDTA‐free (25×). Before centrifugation at 15,250 ×*g* for 10 min. at 4°C, the cell extract was sonicated to shred remaining cellular components.

A total of 25 μg of whole or fractionated cell protein lysates were separated by SDS‐PAGE under reducing conditions using Mini‐Protean TGX stain‐free precast gels (4–15% or 10% resolving gels, Bio‐Rad Laboratories, Munich, Germany) and then transferred onto nitrocellulose membranes (GE Healthcare, Buckinghamshire, UK) in a semidry apparatus. Protein was imaged by UV transillumination after activation using Chemidoc XRS (Bio‐Rad Laboratories). Membranes were blocked with RotiBlock (Roth, Karlsruhe, Germany) for 1 hr at room temperature (RT) followed by incubation with the primary rabbit anti‐ATP6AP2 antibody (1:2000; Sigma‐Aldrich, Munich, Germany) overnight. Protein expression was visualized using a horseradish peroxidase (HP)‐conjugated secondary anti‐rabbit antibody and enhanced chemiluminescence reagent (Bio‐Rad Laboratories). Images were obtained using an image capture system (Chemidoc XRS, Bio‐Rad Laboratories) and quantified for band intensity using Image Lab software (Bio‐Rad Laboratories). All experiments were performed six times. Whole protein was used as loading control, and the PageRuler Prestained Protein Ladder (Thermo Fisher Scientific Inc, Germany) served as molecular weight marker.

### Transcriptome analyses

For microarray‐based transcriptome analyses, total RNA was extracted by a modified phenol extraction protocol using Trizol reagent (Thermo Fisher Scientific, Invitrogen, Germany) as described previously 16. Total RNA was further purified using the RNA Clean‐Up and Concentration Micro Kit (Norgen, Thorold, Ontario, Canada), and concentrations were measured using a ND‐1000 spectrophotometer (Thermo Fisher Scientific Inc, Wilmington, DE, USA). The integrity of the RNA preparations was validated by means of lab‐on‐chip capillary electrophoresis technology (Bioanalyzer 2100; Agilent Technologies, Santa Clara, CA, USA). Only RNA samples fulfilling the following criteria were used for the transcriptome analyses: RNA Integrity Number (RIN) >7.5 28, A_260 nm/280 nm_ ≥1.8, A_260 nm/230 nm_ ≥1.9. Individual RNA samples prepared from untreated or treated As4.1 cells (*n* = 3 each) were subjected to transcriptome analyses using GeneChip Mouse Gene 1.0 ST arrays (Affymetrix Inc, Santa Clara, CA, USA). Target preparation and array hybridization were performed according to the manufacturer's instructions using the AMBION WT Expression Kit and GeneChip WT Terminal Labeling and Controls Kit (Life Technologies Inc and Affymetrix Inc). Quality assessment of all hybridizations was carried out by inspecting scan images and by carefully reviewing external and endogenous controls using the Expression Console software (Affymetrix Inc). For all processed arrays, the available control parameters passed the default threshold tests and all arrays were considered to be of good quality.

Microarray data were analysed using the Rosetta Resolver^®^ system (Rosetta Biosoftware, Seattle, WA, USA) by processing the Affymetrix CEL files using the Affymetrix Rosetta intensity data summarization. In brief, normalized intensity signals were calculated by processing the Affymetrix CEL files using the Affymetrix Rosetta intensity data summarization. The raw data were background corrected, log2‐transformed and quantile‐normalized. Normalized expression values for each transcript/probe set were calculated by a summarization of multiple probe intensities for each probe set, based on a multi‐array model fit robustly using the median polish algorithm. Samples were analysed based on fold change calculations and signal statistics after direct comparisons of different samples. Genes exhibiting significantly different expressions on the mRNA level were identified using the following cut‐off criteria: one‐way anova with Benjamini and Hochberg FDR (*P* ≤ 0.05), signal correction statistics (Ratio Builder, *P* ≤ 0.05) and fold change ≥1.5‐fold.

### Quantitative PCR and PCR Array

For qRT‐PCR and PCR arrays, RNA was extracted using the RNeasy Mini Kit (Zymo Research, Freiburg, Germany) according to the manufacturer's instructions. Quality was checked by spectrophotometry (DS‐11+, DeNovix Inc, Wilmington, DE, USA). RNA was reverse transcribed to cDNA (High Capacity cDNA Kit; Life Technologies), which was then stored at −70°C.

For qRT‐PCR, cDNA was diluted in nuclease‐free water and duplicates of 20 ng reactions were performed with SYBR^®^ FAST Universal 2× Master Mix containing SYBR green dye and optimized primer pairs: ATP6AP2: FOR: *TGGGAAGCGTTATGGAG AAG,* REV: *CTTCCTCACCAGGGATGTGT*; Tyrosine 3‐Monooxygenase/Tryptophan 5‐Monooxygenase Activation Protein, Zeta (YWHAZ): FOR: *CATCTGCAACGACGT ACTGTCTCT,* REV: *GACTGGTCCACAATTCCTTTC TTG*. The threshold cycle number (CT) in combination with the 2^−∆∆CT^ method was normalized against *YWHAZ* and compared to the control. Transcript levels of selected genes were validated using a custom mouse RT^2^Profiler™ PCR Array (CAPM12033; Qiagen, Venlo, the Netherlands) according to the manufacturers' instructions.

### Immunocytochemistry and fluorescence microscopy

After removal of medium and three washings in PBS, cells were fixed in 2% paraformaldehyde for 10 min. and permeabilized in 0.3% triton X‐100 in PBS for 5 min. at RT. Non‐specific binding sites were blocked by incubating cells in blocking solution containing 2% foetal bovine serum, 2% foetal bovine serum fraction V and 0.2% fish gelatine for 1 hr at RT. Samples were washed three times in PBS and then incubated for 1 hr with the primary mouse anti‐acetylated α‐tubulin antibody (1:500; Sigma‐Aldrich) or the rabbit anti‐ATP6AP2 antibody (1:500; Sigma‐Aldrich), or the mouse anti‐PDI antibody (1:500; Thermo Scientific Inc, Germany). Cells were washed again three times in PBS and incubated with the corresponding 1:500 diluted secondary antibodies (Alexa Fluor 488 chicken anti‐mouse and Alexa Fluor 596 donkey anti‐rabbit) for 1 hr at RT. Cells were finally washed three times in PBS and mounted onto glass slides for 24 hrs at 4°C using fluorescent mounting medium containing DAPI (DAKO Omnis, Hamburg, Germany). Slides were imaged using a fluorescence microscope (BZ 9000; Keyence Corp, Osaka, Japan) with a 40× or a 100× plan apochromat oil‐immersion objective. Primary cilia lengths were measured using IMAGE J software.

### Proliferation rate and cell cycle analyses

Proliferation of As4.1 cells was measured using the Cell Proliferation ELISA (Roche Applied Science, Mannheim, Germany) according to the manufacturer's protocol. Briefly, 1 × 10^4^ pretreated cells/well/100 μl medium were seeded in 96‐well plates as triplicates, cultured for 4 hrs at 37°C and 5% CO_2_ and labelled with BrdU solution for 20 hrs. After fixation and denaturation, cells incorporating BrdU into the DNA during proliferation were labelled by adding an anti‐BrdU‐POD antibody for 90 min. at RT followed by extensive washes in washing buffer. Addition of POD substrate for 10 min. at RT in the dark was terminated by adding 25 μl of 1 mol/l H_2_SO_4_. Then, cells were measured immediately at 450 nm using a plate reader (MRX; Dynatech Laboratories Inc, San Francisco, CA, USA).

For cell cycle analyses, 1 × 10^6^ pretreated cells were harvested, washed twice with cold PBS and fixed in 70% ethanol at −20°C for 2 hrs. After two washings with PBS, cells were resuspended in 1 ml PBS containing 50 μg/ml RNase A (Sigma‐Aldrich). Following an incubation period of 1 hr at 37°C to degrade RNA and a wash step, cells were loaded with 50 μg/ml propidium iodide (PI) for 10 min. in the dark at RT. The percentage of cells with different DNA contents corresponding to different phases of the cell cycle was measured by flow cytometry (FACS Calibur, BD).

### Determination of lysosomale H^+^ content and cell death

Twenty‐four hour after siRNA transfection or bafilomycin A exposure, adherent cells were detached from the culture dishes by trypsin/EDTA treatment. Supernatants containing floating, dead cells were collected and reunited with detached adherent cells. After centrifugation, cell pellets were resuspended in Annexin binding buffer. Cells were counted and apoptosis was determined by Annexin V labelling (BD Pharmingen, Heidelberg, Germany). For this purpose, 10^5^ cells were incubated for 15 min. at RT in the dark in 100 μl binding buffer containing 5 μl Annexin V. Then, unbound Annexin V was removed by washing the cells with 3 ml binding buffer. Caspase activity was determined by incubation cells with 5 μl of the 1:100 diluted CaspACE FITC‐VAD‐FMK dye (Promega, Mannheim, Germany) for 20 min. at 37°C. The excess of dye was removed by washing the cells with 3 ml FACS buffer. Before measurement, cells were additionally incubated with 500 ng/ml PI for 5 min. to discriminate between apoptotic and necrotic cells.

To detect lysosomale H^+^ concentration, the acidotropic dye LysoTracker Red DND‐99 (Thermo Fisher Scientific, Invitrogen) was used according to manufacturer's instructions. In brief, 10^5^ cells were incubated in 500 μl culture medium containing 0.5 μl LysoTracker dye (final concentration 1 μM) for 30 min. at 37°C in the dark. Then, unbound dye was removed by washing the cells with 3 ml culture medium. Resuspended cells were analysed immediately by recording the mean fluorescence intensity (FLI) of LysoTracker‐positive cells.

Data from 5000 cells were analysed on a FACS Calibur flow cytometer (BD). Cell debris was excluded from the measurement by setting a gate for intact cells. The data were analysed by Cell Quest software (BD Biosciences, Heidelberg, Germany).

### Statistical analyses

All analyses were performed using Excel and GRAPHPAD PRISM (La Jolla, CA, USA). Student's *t*‐test was performed to compare mean values between two groups. For comparisons among three or more groups, one‐way or two‐way anova, respectively, followed by the Bonferroni *post hoc* test, was used. Data are presented as mean ± S.E.M.

## Results

### Knock‐down of *ATP6AP2* by siRNA is efficient 12–72 hrs after transfection

**Figure 1:**
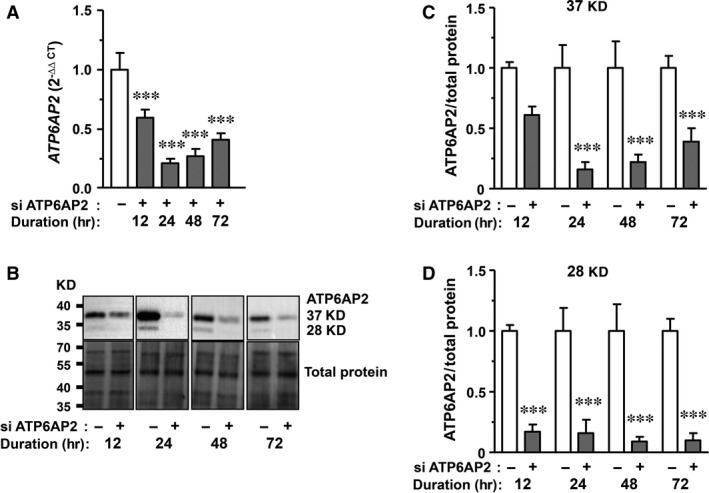
ATP6AP2 expression is efficiently down‐regulated by siRNA interference. Efficacy of *ATP6AP2* knock‐down using 40 nM siRNA was checked time dependently by (**A**) qRT‐PCR normalized to the housekeeping gene *YWHAZ* and the scramble control [*ATP6AP2* (2^−∆∆CT^)] (*n* = 9) as well as by (**B** and **C**) Western blotting normalized to total protein content (*n* = 5). (**B**) Representative Western blots depict the protein bands at 37 and 28 kD corresponding to the full‐length and the soluble ATP6AP2. (**C** and **D**) Quantification of protein bands at 37 and 28 kD related to total protein. ***P < 0.001 *versus* controls.

To identify the optimal time‐point for functional analyses, expression of *ATP6AP2* was determined 12–72 hrs after siRNA application. Efficiency and time course of knock‐down were analysed by RT‐PCR and Western blot. Reductions in *ATP6AP2* transcript levels to about 59%, 21%, 27% and 41% were seen at 12, 24, 48 and 72 hrs after transfection, respectively. This indicates an efficient siRNA‐mediated *ATP6AP2* knock‐down at 24 and 48 hrs followed by a gradual loss of efficacy at later time‐points (Fig. 1A).

Western blot analyses revealed protein bands at 37 and 28 kD corresponding to the expected full‐length and truncated soluble *ATP6AP2*, respectively (Fig. 1B–D). Efficient knock‐down of *ATP6AP2* protein levels by siRNA interference was quantified at the indicated time‐points, too, with respect to both the 37 and the 28‐kD bands.

### The toxicity of *ATP6AP2* knock‐down is mild and less pronounced than that of bafilomycin A

**Figure 2:**
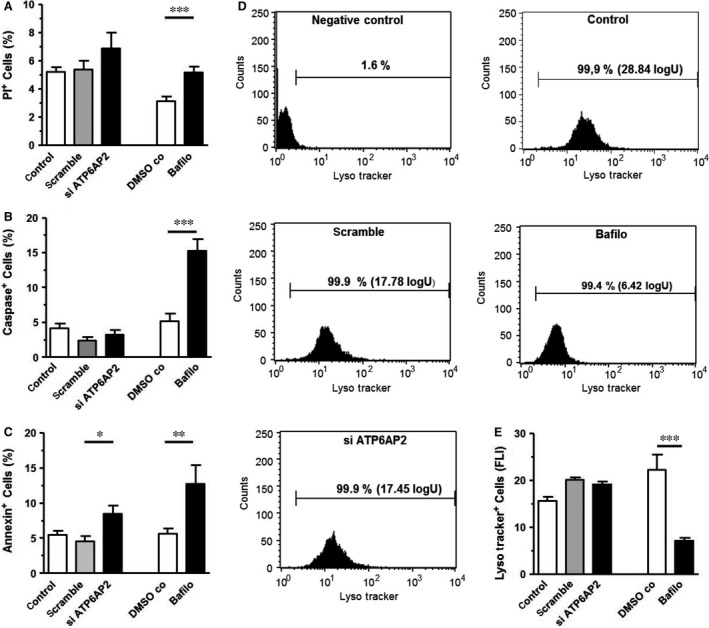
Cell death induced by *ATP6AP2* knock‐down and V‐ATPase inhibition related to acidification of lysosomal compartments. (**A**) Viability of the cells as checked by propidium iodide labelling (*n* = 14). (**B** and **C**) Percentage of apoptotic annexin V‐positive cells (*n* = 9) and caspase‐positive cells (*n* = 8) determined by flow cytometry. (**D**) Representative FACS analyses of LysoTracker‐positive cells pretreated for 24 hrs by scramble siRNA, by siRNA to *ATP6AP2* or by bafilomycin (1 μmol/l). (**E**) Quantitative FLI data of LysoTracker‐positive cells (*n* = 6). ***P < 0.001; **P < 0.01 and *P < 0.05 *versus* controls.

To detect functional consequences of *ATP6AP2* knock‐down, we analysed the cells 24 hrs after transfection. The viability of *ATP6AP2*‐depleted cells assessed by PI staining was not compromised (Fig. 2A). The number of caspase‐positive cells was also not impaired by *ATP6AP2* knock‐down (Fig. 2B). Using the apoptosis marker annexin V, we observed significantly increased apoptotic cell death rates (Fig. 2C). The fraction of annexin V‐positive cells rose to 8.44 ± 1.21% in *ATP6AP2*‐depleted cells compared to 5.48 ± 0.56% and 4.53 ± 0.80% in untreated and scramble controls, respectively.

Bafilomycin A treatment, which inhibits V‐ATPase functions, resulted in a moderate increase in PI‐positive cells from 3.1 ± 0.3% to 5.1 ± 0.4%, a doubling of annexin V‐positive cells (5.6 ± 0.7% to 12.7 ± 2.7%) and a threefold increase in caspase‐positive cells from 5.2 ± 1.0% to 15.3 ± 1.6% (Fig. 2A–C).

### Bafilomycin A treatment but not *ATP6AP2* down‐regulation causes deacidification of lysosomal compartments

The inhibition of V‐ATPase activity was confirmed by the observation of markedly decreased LysoTracker staining by bafilomycin A. Because V‐ATPase is involved in lysosomal acidification, inhibition of V‐ATPase should prevent the passage of protons into lysosomes. Indeed, using the acidotropic dye LysoTracker to stain cellular acidic compartments, we observed a marked decrease in the FLI of LysoTracker‐positive cells from 22.26 ± 3.24 logU in DMSO controls to 7.05 ± 0.62 logU in bafilomycin‐treated cells (Fig. 2D and E). In contrast, 24‐hrs *ATP6AP2* knock‐down did not influence acidity of lysosomal compartments, because FLI remained stable in *ATP6AP2*‐depleted cells (19.61 ± 0.62 logU) compared to the corresponding controls (15.63 ± 0.82 and 20.07 ± 0.60 logU) (Fig. 2D and E).

### The expression pattern downstream of ATP6AP2 identifies genes associated with the primary cilium

To obtain further insights into the mechanisms how the knock‐down of *ATP6AP2* affects the cell cycle, we performed a transcriptome analysis downstream of *ATP6AP2* knock‐down using GeneChip Mouse Gene 1.0 ST array (Affymetrix). As expected, *ATP6AP2* was down‐regulated 2.6‐fold. Validation by anova analyses of variance and by setting a minimally altered significance threshold of 1.5‐fold restricted the number of differentially expressed genes to 30. These genes are listed in Table 1 and classified according to different pathways. In this context, we were able to identify several transcripts which are associated with the primary cilium and involved in cell cycle regulation. Some of these transcripts were categorized as Bardet–Biedl Syndrome (*Bbs*)‐ and Meckel–Gruber Syndrome (*Mks*)‐associated genes causing ciliopathies.

**Table 1:** Transcripts identified downstream of ATP6AP2/(P)RR 24 hrs after transfection

Sequence ID	Gene symbol	Protein name	*P*‐value	Fold change	Chrom	Localization	Ref.
1124131	*Atp6ap2*	ATPase, H^+^ transporting lysosomal accessory protein 2	0.030	−2.57	X	Membrane, ER	
Primary cilia/flagella							
1129411	*Rabl5 (Ift22* a *)*	RAB, member of RAS oncogene family‐like 5	0.012	2.13	5	Ift complex B	39
1102442	*Ttc26 (Dyf13* a *)*	Tetratricopeptide repeat domain 26	0.003	1.79	6	Ift complex B	40
1130399	*4932417I16Rik (Tmem231* a *; Mks11* a *)*	RIKEN cDNA 4932417I16 gene	0.007	1.91	8	TZ	41
1136082	*Rpgrip1l (Mks5* a *, Nphp8* a *)*	Rpgrip1‐like	0.041	1.78	8	TZ, basal body	29, 42
1123486	*Tctn2*	Tectonic family member 2	0.029	1.78	5	TZ	43
1105543	*Tmem216 (Mks2* a *)*	Transmembrane protein 216	0.046	1.51	19	TZ, basal body	44
1120695	*Bbs1*	Bardet–Biedl syndrome 1 (human)	0.017	1.70	19	BBSome	45
1130375	*Bbs7*	Bardet–Biedl syndrome 7 (human)	0.027	1.52	3	BBSome, centrosome	46
1135586	*Arl6 (Bbs3* a *)*	ADP‐ribosylation factor‐like 6	0.026	1.55	16	BBSome, cytosol	47
1112414	*Nme7*	Non‐metastatic cells 7, protein expressed in (nucleoside‐diphosphate kinase)	0.038	1.65	1	γ‐tubulin ring Complex, centrosome	44
Cell cycle							
1117889	*1700007K13Rik (Pierce1, RbEST47* a *)*	RIKEN cDNA 1700007K13 gene	0,043	2.20	2	Unknown	19
1110632	*Ppif (CypD* a *)*	Peptidylprolyl isomerase F (cyclophilin F, D)	0.032	1.57	14	Mitochondria, cytosol	48
1129925	*Clock*	Circadian locomoter output cycles kaput	0.029	−1.67	5	Cytosol, nucleus	49
Cell migration/cytoskeleton							
1105470	*Slit3*	Slit homologue 3 (Drosophila)	0.009	−1.66	11	Secretion	50
1136195	*A230083H22Rik (Prune2, Bmcc1* a *)*	RIKEN cDNA A230083H22 gene	0.048	−1.65	19	Cytosol, endosomes, microtubuli	51, 52
Secretory pathway/traffick							
1124988	*Abcb6*	ATP‐binding cassette, subfamily B (MDR/TAP), member 6	0.020	1.57	1	ER, endo‐, exosomes	53
1108606	*Acsl4*	Acyl‐CoA synthetase long‐chain family member 4	0.007	−1.61	X	ER, lysosomes	54
1133354	*Lrrc8b*	Leucine‐rich repeat containing 8 family, member B	0.023	−1.78	10	Membrane, VRAC	55
1103969	*L7Rn6*	Lethal, Chr 7, Rinchik 6	0.042	−1.95	7	ER, cytosol, nucleus	56
Extracellular matrix							
1130315	*Mmp19*	Matrix metallopeptidase 19	0.040	−1.75	10	Secretion	57
Transcription/translation							
1136275	*Tarbp2*	TAR (HIV) RNA‐binding protein 2	0.035	1.55	15	Cytosol, nucleus	58
1133367	*Dalrd3*	DALR anticodon binding domain containing 3	0.045	1.65	9	Cytosol, nucleus	59
1120276	*Fkbp3 (Fkbp25* a *)*	FK506‐binding protein 3	0.013	−1.84	12	ER, cytosol, nucleus	60
Apoptosis							
1116780	*LOC100045562*	PREDICTED: Mus musculus similar to ornithine decarboxylase (LOC100045562), misc RNA.	0.034	1.67	7	Cytosol; ER, Golgi	61
1119394	*Ube2v2 (Mms2* a *)*	Ubiquitin‐conjugating enzyme E2 variant 2	0.048	−1.54	16	Cytosol	62
Wnt/ß‐Catenin pathway							
1122967	*Cyp1a1*	Cytochrome P450, family 1, subfamily a, polypeptide 1	0.025	2.19	9	Cytosol, microsomes	63
1126392	*Fibin*	Fin bud initiation factor homologue (zebrafish)	0.046	−2.49	2	ER, Golgi, secretion	64
Unknown protein/function							
1109622	*AI429214 (C8orf48* a *)*	Expressed sequence AI429214	0.042	2.01	8		
1131132			0.048	1.72			
1110402			0.015	−1.50			

The sequence ID, primary sequence name (gene symbol), anova statistic, fold changes and the chromosomal and protein localization are listed for each corresponding protein linked to a signalling pathway. Data represent three experiments with statistical analysis according to anova *post hoc* test.

a Indicates alternative name.

Chrom: Chromosome; Ref: References; Bbs: Bardet–Biedl Syndrome; Mks: Meckel–Gruber Syndrome; Ift: intraflagellar transport; TZ: transition zone; ER: endoplasmatic reticulum; VRAC: volume‐regulated anion channel.

**Figure 3:**
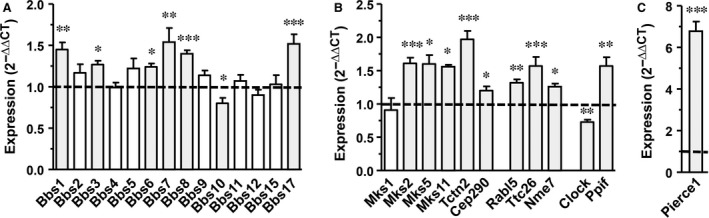
*ATP6AP2* down‐regulation changes expression pattern of transcripts associated with primary cilia and cell cycle. PCR array data of As4.1 cells pretreated with scramble siRNA or with siRNA to *ATP6AP2* (each 40 nmol) for 24 hrs. Expression data (*n* = 6) were normalized to the housekeeping gene *YWHAZ* and to the scramble control (2^−∆∆CT^). ***P < 0.001; **P < 0.01; *P < 0.05 *versus* scramble control.

To further corroborate the above Affymetrix analysis data, we designed a custom mouse RT profiler PCR array to investigate the transcript levels of 14 BBS‐ and six MKS‐associated proteins and eight proteins assignable directly to primary cilia or to the cell cycle. By focussing on these few selected genes and by analysing six samples per group, we markedly increased the statistical power. Of the ciliary Bbs genes, *Bbs1, Bbs3, Bbs6, Bbs7, Bbs8, Bbs10 and Bbs17* exhibited differential expression, while *Bbs2, Bbs4, Bbs5, Bbs9, Bbs11, Bbs12 and Bbs15* transcript levels were not modified by *ATP6AP2* knock‐down (Fig. 3A). With the exception of *Bbs10,* all other regulated Bbs genes were up‐regulated. The group of tested ciliary Mks‐related genes included *Tctn2, Tmem231/Mks11, Tmem216/Mks2, Rpgrip1l/Mks5, Cep290* and *Mks1*. The *Mks1* transcript level was not influenced by *ATP6AP2* down‐regulation, whereas all other transcript levels were up‐regulated 24 hrs after transfection (Fig. 3B). A similar up‐regulation was observed for *Rabl5/Ift22*,* Ttc26/Dyf13* and *Nme7* transcript levels, whose proteins are also related to the primary cilium (Fig. 3B).

Regarding the tested cell cycle‐associated genes, *Pierce1/RbEST47* responded with a prominent 6.8‐fold increase in its transcript level, 24 hrs after transfection (Fig. 3C). *Ppif* expression was increased 1.6‐fold, whereas C*lock was* 1.4‐fold down‐regulated at this time‐point (Fig. 3B).

### Effects of ATP6AP2 knock‐down on the expression pattern: no indication of V‐ATPase dependency

As documented in numerous studies, several functions of *ATP6AP2* are mediated by interaction with the V‐ATPase. To differentiate between V‐ATPase‐dependent and V‐ATPase‐independent gene expression patterns downstream of *ATP6AP2*, As4.1 cells were treated with the V‐ATPase inhibitor bafilomycin A for 24 hrs. Overall, 626 genes were differentially regulated in bafilomycin‐treated cells compared to control cells (data not shown). The expression pattern included a 2.22‐ and a 2.05‐fold up‐regulation of the *V0 subunit B* and the *V1 subunit B2* of the H^+^ transporting lysosomal ATPase and a 2.05‐fold up‐regulation of the *ATP6AP2* transcript level. The majority of affected genes did not match between bafilomycin‐treated and *ATP6AP2* knock‐down cells. The expression of only 34 transcripts was differentially regulated by each of the two interventions, however, most of them in the opposite direction (Table 2). Of special interest, bafilomycin treatment did not affect the transcript levels of cilia‐related genes indicating that the effects of *ATP6AP2* knock‐down on the cell cycle are likely V‐ATPase‐independent.

**Table 2:** Transcripts regulated by both siRNA‐mediated ATP6AP2 knock‐down and V‐ATPase inhibition

Sequence ID	Gene symbol	Protein name	Fold change	
			siRNA	Bafi
1124131	*Atp6ap2*	ATPase, H^+^ transporting, lysosomal accessory protein 2	−2.57	2.51
1134827	*Acta2*	Actin, α 2, smooth muscle, aorta	2.58	−4.68
1115329	*Alpl*	Alkaline phosphatase, liver/bone/kidney	1.51	−3.16
1123891	*Atp1b1*	ATPase, Na+/K+ transporting, β 1 polypeptide	1.69	−2.34
1123050	*Bmp4*	Bone morphogenetic protein 4	2.06	−2.60
1122659	*Car9*	Carbonic anhydrase 9	−1.77	3.22
1131781	*Cav1*	Caveolin 1, caveolae protein	1.68	−4.83
1134774	*Ccl2*	Chemokine (C‐C motif) ligand 2	2.20	−4.16
1129352	*Chac1*	ChaC, cation transport regulator‐like 1 (*E. coli*)	2.47	1.99
1125858	*Dpp7*	Dipeptidylpeptidase 7	1.78	2.74
1132231	*Enpep*	Glutamyl aminopeptidase	−1.68	−3.47
1112069	*Figf*	c‐fos‐induced growth factor	2.07	−1.95
1109935	*Gadd45a*	Growth arrest and DNA damage‐inducible 45 α	−3.40	2.53
1134045	*Hspa1a*	Heat shock protein 1A	1.86	−2.23
1109017	*Hspa1b*	Heat shock protein 1B	1.54	−2.43
1135433	*Hist1h1a*	Histone cluster 1, H1a	−1.38	−1.95
1119748	*Irgm1*	Immunity‐related GTPase family M	1.93	−2.18
1133764	*Igfbp4*	Insulin‐like growth factor‐binding protein 4	−1.54	−2.80
1108676	*Lgi2*	Leucine‐rich repeat LGI family, member 2	−1.82	−1.97
1105408	*Ly6d*	Lymphocyte antigen 6 complex, locus D	−1.73	−4.51
1120944	*Mest*	Mesoderm specific transcript	1.91	−7.92
1112577	*Mt2*	Metallothionein 2	3.95	5.53
1104559	*Nfkbia*	Nuclear factor of kappa light polypeptide gene enhancer in B‐cells inhibitor, α	1.62	−2.30
1117142	*P2rx3*	Purinergic receptor P2X, ligand‐gated ion channel, 3	−5.34	−2.86
1114155	*Padi2*	Peptidyl arginine deiminase, type II	−2.00	−2.87
1118509	*Pi15*	Peptidase inhibitor 15	2.09	−4.63
1130399	*4932417I16Rik*	RIKEN cDNA 4932417I16 gene	1.91	2.16
1111109	*B430001I08Rik_1*	RIKEN cDNA B430001I08 gene	1.53	−2.77
1127478	*S100g*	S100 calcium‐binding protein G	1.74	−2.60
1105372	*Tapbp*	TAP‐binding protein	1.55	1.86
1110002	*Thbs2*	Thrombospondin 2	−1.76	−3.41
1122671	*Tnfsf15*	Tumour necrosis factor (ligand) superfamily, member 15	−2.10	−7.79
1112165	*Tpp1*	Tripeptidyl peptidase I	1.52	2.03
1119180	*Vtcn1*	V‐set domain containing T‐cell activation inhibitor 1	3.39	−2.28

The sequence ID, primary sequence name (gene symbol) and fold changes are listed for each corresponding protein. Data represent one and three experiments, respectively, with statistical analyses according to *t*‐test and a minimum of 1.5‐fold changes.

### *ATP6AP2* knock‐down increases the proportion of cells carrying a primary cilium

**Figure 4:**
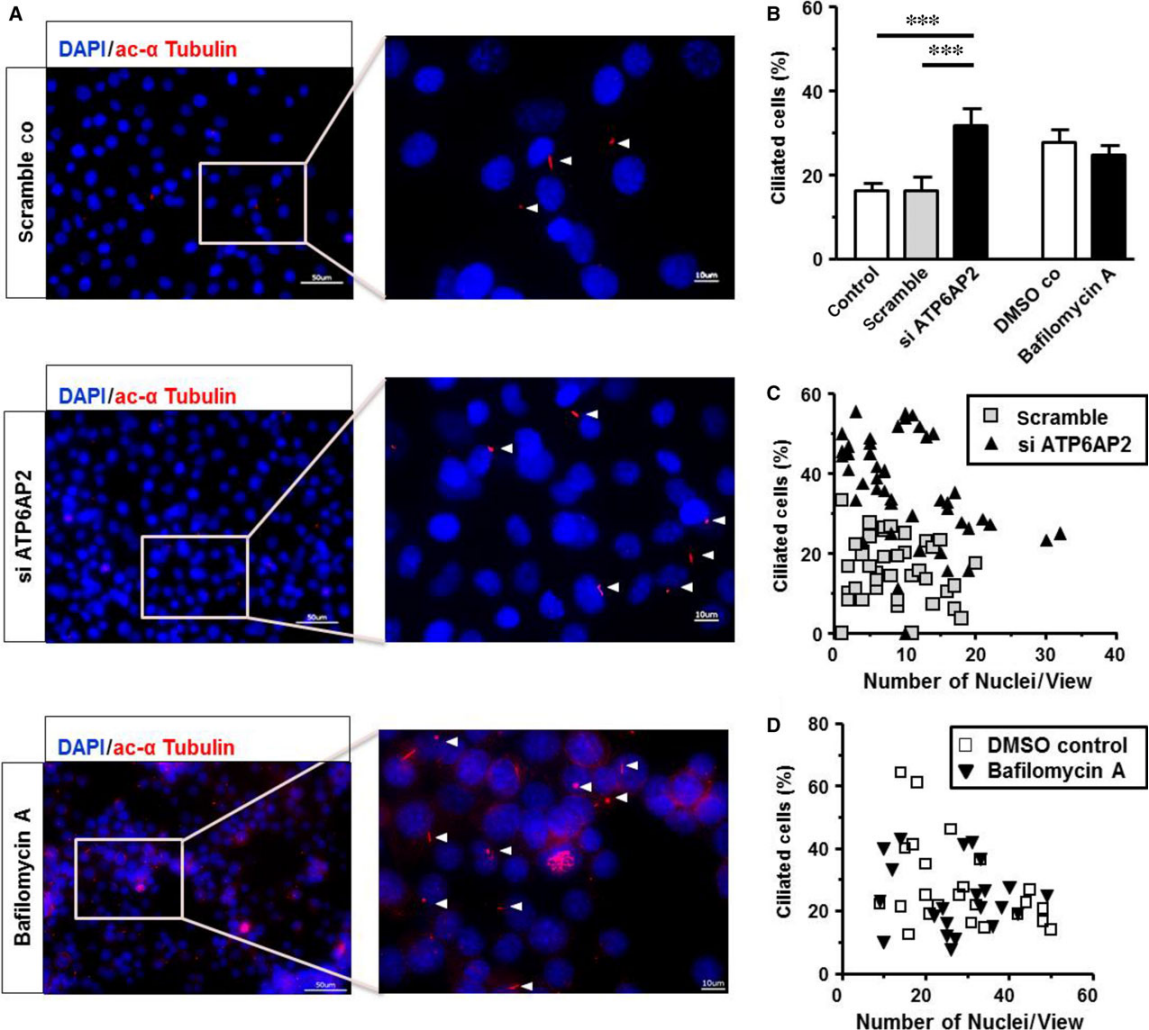
*ATP6AP2* knock‐down enhances the expression of the primary cilium. (**A**) Representative fluorescence microscopic images of scramble controls (upper panel), *ATP6AP2*‐depleted cells (central panel) and bafilomycin‐treated cells (lower panel). Primary cilia (red) were probed with the anti‐ac‐α‐tubulin antibody. 4′,6′‐Diamidino‐2‐phenylindole (DAPI, blue) was used for labelling the cell nucleus. Bars represent an size of 50 and 10 μm. (**B**) Percentage of cells carrying a primary cilium *n* = 4; ***P < 0.001 *versus* control groups. (**C** and **D**) Correlation between the number of nuclei per field of vision and percentage of ciliated cells after ATP6AP2 knock‐down or bafilomycin treatment (data from *n* = 3 independent experiments).

In this study, *ATP6AP2* knock‐down was associated with the doubling of the percentage of ciliated cells (Fig. 4A). Specifically, the percentage of ciliated cells related to the total number of cell nuclei increased significantly from 16.2 ± 1.8% and 16.4 ± 3.2% in untreated and scramble controls, respectively, to 31.8 ± 4.0% in *ATP6AP2*‐depleted As4.1 cells. Considering that the cell density on the slide may influence the number of ciliated cells due to a contact‐induced cell cycle arrest within the G1 phase, we correlated the percentage of ciliated cells with the number of nuclei per field of vision (Fig. 4B). Our data demonstrate that the cell density of scramble controls and *ATP6AP2*‐depleted cells on the slides was low enough to avoid contact‐induced cell cycle arrest.

Ciliary length was not influenced in *ATP6AP2*‐depleted As4.1 cells compared to controls. Ciliary lengths amounted to 2.84 ± 0.94 μm in scramble controls (*n* = 37) and 2.81 ± 1.03 μm in *ATP6AP2*‐depleted As4.1 cells (*n* = 45) (data not shown). Furthermore, we did not observe cells with more than one primary cilium or with a cilium with altered length. Therefore, we exclude dysregulated ciliogenesis.

Treatment of cells by bafilomycin A influenced neither the percentage of ciliated cells (Fig. 4A and C) nor the ciliary length compared to DMSO‐treated controls (data not shown).

### *ATP6AP2* down‐regulation inhibits proliferation and causes cell cycle arrest

**Figure 5:**
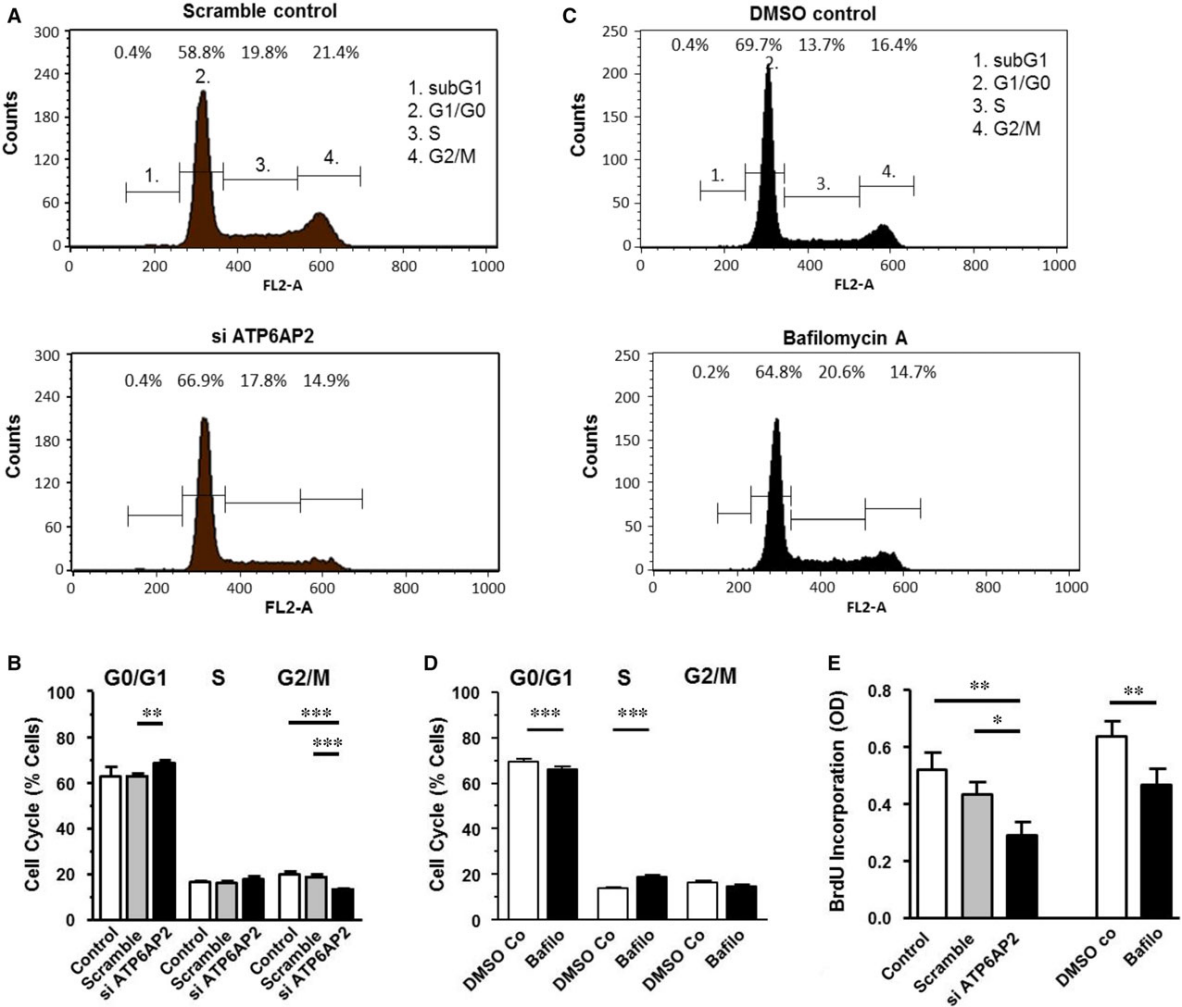
*ATP6AP2* knock‐down and V‐ATPase inhibition cause restriction of proliferation and cell cycle arrest at different phases. (**A** and **C**) Representative cell cycle analyses of scramble controls, of *ATP6AP2*‐depleted cells 24 hrs after transfection and of bafilomycin‐treated cells. (**B** and **D**) Proportion of cells associated with different cell cycle phases (*n* = 8). (**E**) Proliferation rate as detected by the BrdU incorporation (*n* = 8). ***P < 0.001; **P < 0.01 and *P < 0.05 *versus* corresponding controls.

Both *ATP6AP2* knock‐down and bafilomycin A decreased proliferation rates (Fig. 5E). However, the effects of *ATP6AP2* knock‐down and of bafilomycin A on the cell cycle were different. We analysed the distribution of cells within the cell cycle stages according to their DNA content. FACS analyses illustrate a significant increase in the number of G0/G1 cells and a decrease of the G2/M fraction in *ATP6AP2*‐depleted As4.1 cells *versus* scramble siRNA‐treated cells 24 hrs after transfection (Fig. 5B). This indicates a reduced number of mitotic cells, an enhanced rate of quiescent cells and a cell cycle arrest between G1 and S phase. The effect seen with respect to the G1 to S transition is likely underestimated, as most cells were already in the G0/G1 cell cycle phase and only 15–20% in the mitotic stages (G2/M).

The effect of *ATP6AP2* knock‐down is not accounted for by inhibition of V‐ATPase activity/acidification, because it cannot be mimicked with bafilomycin A. Bafilomycin‐pretreated cells showed a decreased percentage of G0/G1 cells and an accumulation of cells in the S phase (Fig. 5C and D). This effect on cell cycle dynamic induced by V‐ATPase inhibition was already observed in a previous report 17.

### ATP6AP2 translocates from the endoplasmatic reticulum to the mitotic spindle apparatus during cell cycle progression

**Figure 6:**
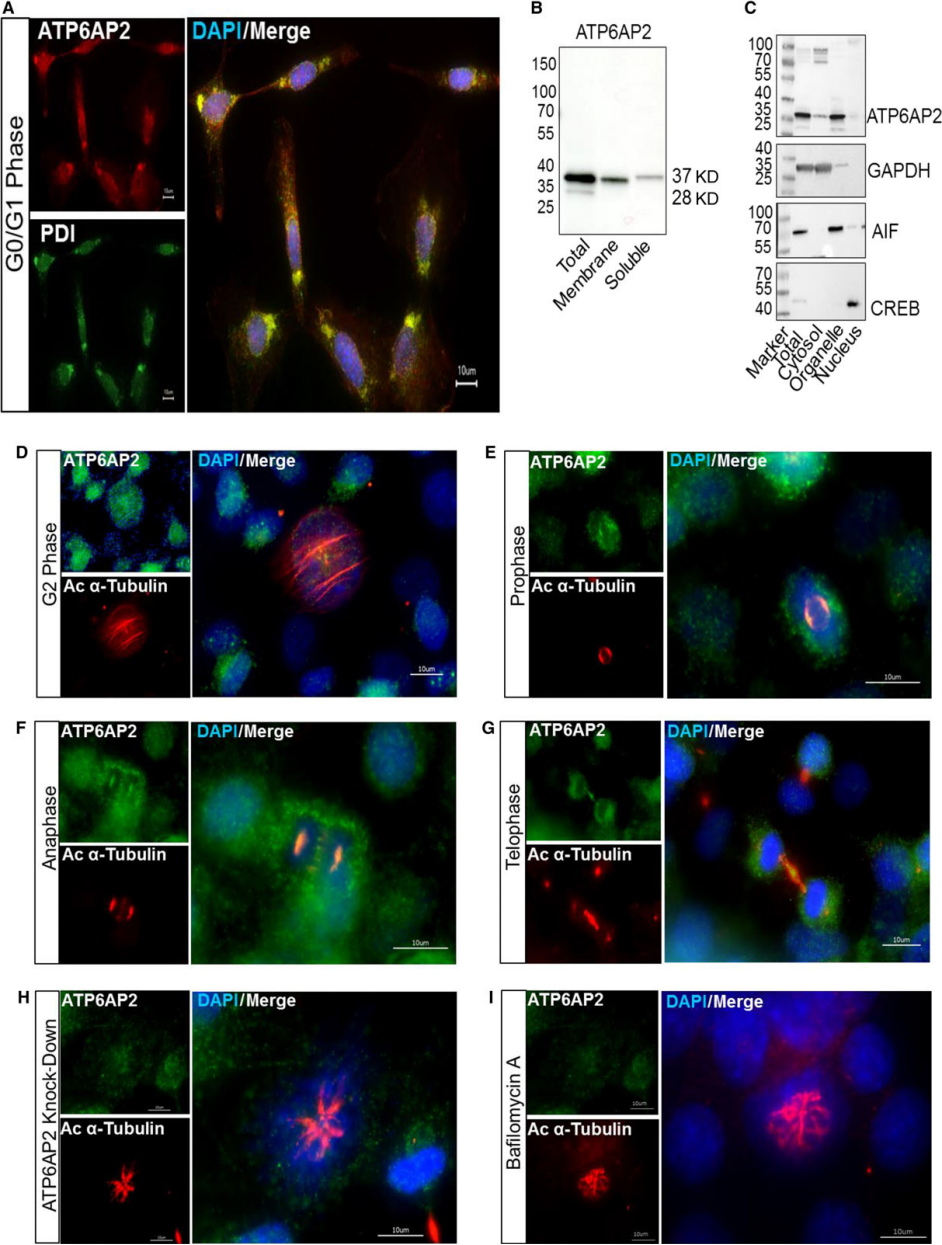
Localization of ATP6AP2 during different cell cycle phases. Representative fluorescence microscopic images of untreated As4.1 cells during different phases of the cell cycle (**A**: G0/G1 phase, **D**: G2 phase, **E**: prophase, **F**: anaphase and **G**: telophase) or of *ATP6AP2*‐depleted cells (**H**) and bafilomycin‐treated cells (**I**) during mitosis. Anti‐PDI antibody (green) was used to mark the ER. Anti‐acetylated α‐tubulin antibody (red) was used for labelling of microtubules (mitotic spindle, midbody). ATP6AP2 distribution was detected with the anti‐ATP6AP2 antibody as indicated. Nuclei were labelled using DAPI (blue). Bars represent an size of 10 μm. (**B** and **C**): Representative Western blots showing the subcellular localization of ATP6AP2 in membrane and soluble fractions (**B**) as well as in different organelle fractions (**C**) verified by antibodies to GAPDH (cytosolic marker), AIF (mitochondrial marker) and CREB (nuclear marker).

In agreement with the current literature 2, 8, ATP6AP2 was located perinuclear and at spots disseminated within the whole cell, suggesting that in As4.1 cells, the receptor protein is located predominantly at the endoplasmatic reticulum (ER). The localization of the protein at the ER was confirmed by ER‐specific labelling of the cells with an antibody directed to the luminal protein disulphide isomerase (PDI) (Fig. 6A). Additionally, ATP6AP2 was apparently located within the cytosol as illustrated by a diffuse labelling of the whole cell. To confirm the cytosolic location of ATP6AP2, we prepared subcellular fractions from our cell line and analysed these by Western blotting (Fig. 6B and C). ATP6AP2 was detectable not only in the membrane fraction, but also in the soluble fraction. In the latter, the ATP6AP2 protein band shifted to a slightly higher molecular weight, suggesting post‐translational modification. To validate the cytosolic localization of ATP6AP2, we separated the cytosol from organelles using digitonin. As expected, ATP6AP2 bands appeared in the total cell extract, in the cell fraction containing different organelles and indeed, albeit to a minor extent, within the cytosolic fraction. In contrast, the nuclear fraction did not contain any ATP6AP2 (Fig. 6C).

Together, the anti‐proliferative effects, the enhanced number of ciliated cells and the overall gene expression pattern after *ATP6AP2* knock‐down indicate a role for ATP6AP2 in cell division. Therefore, we investigated those cells that were just about to divide. Surprisingly, during cell division, the ATP6AP2 protein co‐localized with microtubules as indicated by co‐staining with an anti‐acetylated α‐tubulin antibody (Fig. 6D–H). The microtubular scaffold is essential for generating both the primary cilium and the mitotic spindle apparatus. In As4.1 cells, tubulin reorganization occurred during the G2 phase as illustrated by the red‐labelled ring systems surrounding the nucleus shown in Figure 6D. We found spotted ATP6AP2 signals near these ring systems. During the progression of mitosis, duplicated centrioles form the mitotic spindle poles. This was clearly seen in cells staged at the pro‐ and anaphases (Fig. 6E and F). Here, ATP6AP2 seems to translocate to the spindle poles. During the telophase, daughter cells remained connected *via* the intercellular bridge formed by the central spindle bundle. Again, ATP6AP2 was detectable within this bridge in the so‐called midbody (Fig. 6G).

While analysing the consequences of *ATP6AP2* knock‐down on the microtubular scaffold using fluorescence microscopy, we encountered only few *ATP6AP2*‐depleted cells that were in the mitotic phase. These cells had a defective spindle apparatus (Fig. 6H), suggesting that ATP6AP2 may be necessary for spindle formation and progression of cell cycle into mitosis. After bafilomycin A treatment, As4.1 cells showed similar defective mitotic spindles as observed in *ATP6AP2*‐depleted cells (Fig. 6I).

## Discussion

Our findings reveal new links between ATP6AP2 and the cell cycle. First, *ATP6AP2* knock‐down resulted in a decrease in the proliferative capacity as well as an increased percentage of cells in the G0/G1 phase. Second, *ATP6AP2* knock‐down was associated with an up‐regulation of several ciliary genes and an increased proportion of ciliated cells. Third, during mitosis, ATP6AP2 translocated from the ER/Golgi apparatus to the mitotic spindle apparatus, whereas *ATP6AP2*‐depleted cells failed to enter the mitotic phase or if did, showed deranged mitotic spindles. The effects on the G1 to S transition and on the primary cilia appear to be independent of acidification and V‐ATPase activity because *ATP6AP2* knock‐down did not influence lysosomal pH. Instead, the effect of *ATP6AP2* knock‐down on the cell cycle may be explained by the fact that ATP6AP2 as linker between LRP6 and V‐ATPase is necessary for the canonical Wnt pathway, and this linker is now missing**.** The question of whether or not ATP6AP2 modulates V‐ATPase activity may be cell type and context specific. In line with our observation in a study by Kissing *et al*., knock‐down of ATP6AP2 expression resulted in a reduced level of the V0 sector of the v‐ATPase but acidification appeared undisturbed 18.

In our study, *ATP6AP2*‐depleted cells exhibited a reduced proliferative capacity as detected by the reduced BrdU incorporation during the DNA duplication phase (S phase). This finding also suggests that there was a cell cycle arrest at the early S phase or the preceding G0/G1 phase. Indeed, the prominent up‐regulation of the cell cycle‐related gene *Pierce1/RbEST47* points towards an arrest at the S stage. Sung *et al*. 19 identified the corresponding gene product RbEST47 as a cell cycle oscillatory protein whose expression increases during progression from the G1 to S phase. However, considering our data of cell cycle analyses in *ATP6AP2*‐depleted cells, the percentage of S phase cells remained unchanged, whereas the fraction of cells in the G0/G1 phase increased and the fraction of cells in the G2/M phase decreased. Therefore, the up‐regulation of Pierce1/RbEST47 in *ATP6AP2*‐depleted cells may represent a late event during the G1 phase.

In agreement with the hypothesis that *ATP6AP2*‐depleted cells suffered a cell cycle arrest at the G0/G1 phase, we not only found an increased percentage of cells that were in this stage but also an up‐regulation of several ciliary genes and an increased proportion of ciliated cells after *ATP6AP2* knock‐down. Assembly of the primary cilium occurs during the G0/G1 phase and at the beginning of the S phase, whereas disassembly occurs during the S/G2 transition when the basal body of the cilium is released to form the centrosome and to function as microtubule‐organizing centre 20. Both steps are known to be mediated by the canonical Wnt pathway 15. Furthermore, the encoded ciliary proteins represent the basic components of the primary cilium like the basal body (BBS3, BBS1, BBS7), the transition zone (TCTN2, MKS11, MKS2, MKS5), the intraflagellar transport machinery (IFT) (RABL5, TTC26) and the centrosome (NME7) indicating an enhanced ciliogenesis.

The observed cell cycle arrest accompanied by enhanced ciliogenesis appeared to be independent of V‐ATPase activity because *ATP6AP2* knock‐down did not change lysosomal pH and bafilomycin A treatment neither influenced the expression pattern of the ciliary genes nor the percentage of ciliated cells.

Besides the classical functions in pH homeostasis, receptor‐mediated endocytosis or protein processing and degradation, V‐ATPases are also integrated in regulation of cell cycle and apoptosis 17, 21. In MCF‐7 cells, the V‐ATPase inhibitors iejimalides A and B induce S phase cell cycle arrest and trigger apoptosis by mechanisms involving mitochondrial depolarization and oxidative stress. McHenry *et al*. 17 postulate that ROS‐caused single‐strand breaks lead to double‐strand breaks and p53‐dependent to an S phase arrest followed by apoptosis. Currently, we cannot confirm the postulated signal cascade, but the effects, the S phase arrest and an increase in apoptosis, were also seen in our experiment after bafilomycin A treatment in As4.1 cells.

With respect to apoptosis, we found differences between bafilomycin A treatment and ATP6AP2 knock‐down. Whereas bafilomycin A induced both the translocation of phosphatidylserine (annexin V labelling) from the inner to the outer side of the membrane and an activation of caspases, knock‐down of *ATP6AP2* triggered only the phosphatidylserine translocation, indicating different mechanisms or origins of apoptosis induction (mitochondrial, lysosomal, extracellular).

Primary cilia transduce extracellular signals into the cell by acting as mechano‐, photo‐ or chemosensors and by participating in signal cascades such as Sonic hedgehog, Wnt, platelet‐derived growth factor receptor, fibroblast growth factor and mammalian target of rapamycin 22, 23, 24, 25. Through affecting these pathways, *ATP6AP2* knock‐down might influence not only the balance between proliferation, differentiation and cilium‐controlled growth, but also a variety of signalling cascades modulating the cell fate. The exact mechanisms of action of ATP6AP2 remain subject of further studies.

Previously, we demonstrated a crucial role for *ATP6AP2* in the differentiation of adult hippocampal stem cells towards the neuronal linage *via* its interaction with the non‐canonical/PCP Wnt pathway 14. In this context, it is known that cilia influence the balance between the canonical and the non‐canonical Wnt pathways by favouring the latter 26. As canonical Wnt signalling mediates ciliary disassembly 27, restriction of this pathway would first lead to an increased proportion of ciliated cells and second would limit cell cycle progression from the G1 to the S phase 15. This is indeed what we have seen after *ATP6AP2* knock‐down in As4.1 cells. Further, support for the involvement of the non‐canonical Wnt/PCP pathway comes from the fact that *Tmem216* 28 and *Rpgrip1l* 29, which encode both ciliary but also essential proteins of the PCP pathway, were up‐regulated in our study. In the non‐canonical Wnt/PCP pathway, the binding of specific Wnt isoforms such as Wnt4, Wnt5a or Wnt11 to Frizzled receptors activates Dishevelled, which is known to be localized to the cilium 30. Once activated, Dishevelled increases intracellular Ca^2+^ levels that activate downstream effectors regulating cytoskeletal rearrangement and PCP, as well as cilia polarity and orientation 31, 32. Indeed, *Wnt4* and *Wnt5* are expressed in As4.1 cells as documented by the data of our transcriptome analysis. Hence, it is also likely that the enhanced ciliogenesis in response to *ATP6AP2* knock‐down may be due to facilitation of the non‐canonical Wnt/PCP pathway 30. This hypothesis still remains to be proven.

The centrosome not only forms the scaffold for the ciliary basal body but also directs the assembly of the bipolar spindle during mitosis. The mitotic spindle apparatus, especially the spindle poles and the central spindle bundle, were exactly the places where we detected ATP6AP2 protein in dividing As4.1 cells. These findings suggest that ATP6AP2 plays a role for the progression of the cell cycle during mitosis by influencing spindle function and/or assembly. Unfortunately, we were unable to characterize the exact function of ATP6AP2 protein during the mitotic phase as our *ATP6AP2*‐deficient cells hardly progressed to this stage. This is in accordance with increased apoptosis rates and concomitant decrease in proliferation rate. The effect on the mitotic spindle may involve V‐ATPase functions, as both *ATP6AP2* knock‐down and bafilomycin A led to similarly deformed spindles. However, at the present we cannot exclude unspecific effects of bafilomycin A, and we do not know which of several bafilomycin targets (V‐ATPase activity, V0 membrane sectors independent of V‐ATPase activity or even other targets such as SERCA 33) are involved in spindle deformation.

How can the atypical localization of ATP6AP2 in the cytosol and at the spindle apparatus be explained? ATP6AP2 is a single‐pass transmembrane protein. These proteins are usually integrated into membranes of the ER, the Golgi apparatus and Golgi‐derived vesicles, as well as into the plasma membrane. During mitosis, the Golgi complex, mitochondria and the ER undergo morphological and positional changes. Schlaitz *et al*. 34 demonstrated that in the metaphase the ER is excluded from chromosomes and the central spindle area, but was enriched at the spindle poles. Thus, in mitotic As4.1 cells, ATP6AP2 located at the spindle poles may originate from the ER. Furthermore, during telophase, daughter cells are connected by an intracellular bridge that is formed by the central spindle bundle and contains a variety of associated proteins 35. Before abscission of daughter cells, either secretory vesicles leaving the trans‐Golgi network or recycled endosomes are transported to the bridge to fuse with the cleavage furrow and to deliver membrane components lastly to finish cytokinesis 36. Because ATP6AP2 is localized in membranes of secretory vesicles and the plasma membrane, it seems possible that ATP6AP2 translocates to the midzone of the central spindle in this way. Finally, Kanda *et al*. 37 identified full‐length ATP6AP2 in both the membrane and the cytosolic fractions. This finding is in agreement with our data and opens the possibility that ATP6AP2 which is present at microtubules may represent a cytosolic form of the protein. Although a cytosolic localization is atypical for a single‐pass transmembrane protein, such a phenomenon has been described before 38. Thus, the ER‐localized tyrosine phosphatase PRL‐1 exhibits a cell cycle‐dependent distribution pattern similar to that of ATP6AP2. Wang *et al*. 38 reported that in non‐mitotic HeLa cells, PRL‐1 is localized in the perinuclear region, whereas in mitotic cells it appeared at the spindle apparatus including the spindle microtubules. These authors attributed the cell cycle‐dependent distribution of PRL‐1 to the C‐terminal prenylation of the protein at Cys^170^. The latter is necessary for localization of PRL‐1 to membrane structures, including the ER. Currently, there is little information about post‐transcriptional modifications of ATP6AP2 in association with its subcellular localization. Nevertheless, the small shift of the ATP6AP2 band, which occurred between membrane and cytosolic protein fractions in our As4.1 cells, is in agreement with a post‐translational modification of the protein.

Together, ATP6AP2 promotes cell cycle progression, mitosis and proliferation and inhibits differentiation and ciliogenesis. Although many details still need to be elucidated, our data suggest that ATP6AP2 is indispensable for cell cycle progression and that the protein prevents cell cycle exit and ciliogenesis, thereby enabling cells to enter differentiation. The novel link between ATP6AP2 and the cell cycle suggests an important role for this protein in stem cell proliferation and differentiation, as well as in tumourigenesis.

## Conflict of interest

The authors declare that they have no conflict of interests.
